# Survival and Characteristics of Bladder Cancer: Analysis of the Malaysian National Cancer Registry

**DOI:** 10.3390/ijerph18105237

**Published:** 2021-05-14

**Authors:** Mohd Nasrullah Nik Ab Kadir, Suhaily Mohd Hairon, Najib Majdi Yaacob, Azizah Ab Manan, Nabihah Ali

**Affiliations:** 1 Department of Community Medicine, School of Medical Sciences, Universiti Sains Malaysia, Kubang Kerian 16150, Malaysia; mnasrullah@student.usm.my; 2 Unit of Biostatistics and Research Methodology, School of Medical Sciences, Universiti Sains Malaysia, Kubang Kerian 16150, Malaysia; najibmy@usm.my; 3 Timur Laut District Health Office, Penang State Health Department, Ministry of Health Malaysia, Georgetown 11600, Malaysia; a_drazizah@moh.gov.my; 4 Malaysian National Cancer Registry Department, National Cancer Institute, Ministry of Health Malaysia, Putrajaya 62250, Malaysia; drnabihah@nci.gov.my

**Keywords:** bladder cancer, cancer survival, cancer registry

## Abstract

Background: Bladder cancer ranked ninth of principal male cancer in Malaysia. This study aimed to evaluate the clinical characteristics and survival of bladder cancer patients in Malaysia. Methods: A retrospective cohort study was conducted by obtaining records in the Malaysian National Cancer Registry. Patients aged 15 years old and above with diagnosis date between 2007 and 2011 were included. Death was updated until 31 December 2016. Five-year observed survival and median survival time were determined by the life table method and Kaplan–Meier estimate method. Results: Among 1828 cases, the mean (SD) age of diagnosis was 64.9 (12.5) years. The patients were predominantly men (78.7%), Malay ethnicity (49.4%) and transitional cell carcinoma (78.2%). Only 14.8% of patients were at stage I. The overall five-year observed survival and median survival time was 36.9% (95% CI: 34.6, 39.1) and 27.3 months (95% CI: 23.6, 31.0). The highest five-year observed survival recorded at stage I (67.6%, 95% CI: 62.0, 73.3) and markedly worsen at stage II (34.3%, 95% CI: 27.9, 40.8), III (25.7%, 95% CI: 18.7, 32.6) and IV (12.2%, 95% CI: 8.1, 16.3). Conclusions: Survival of bladder cancer patients in Malaysia was lower with advancing stage. The cancer control programme should be enhanced to improve survival.

## 1. Introduction

Bladder cancer ranks as the tenth most frequently diagnosed cancer globally in 2018. It is the ninth most common cancer among men in Malaysia. The incidence of bladder cancer dramatically increases after 65 years old [1]. It is expected to rise with the ageing population [2,3]. Malaysia is projected to have 14.5% of the population aged 65 years old and above in 2040. A marked upsurge from 5% in 2010 [4]. Bladder cancer refers to a malignant tumour, which occurs when cells in the urinary bladder divide uncontrollably, capable of invading surrounding tissues and spreading to other parts of the human body. Nearly all cases of bladder cancer are transitional cell histology, also known as urothelial carcinoma. Other uncommon tissue morphologies are adenocarcinoma, squamous cell carcinoma and sarcoma [5,6,7]. A previous study in Malaysia’s academic centre described that most bladder cancer patients were male, Chinese and morphologically transitional cell carcinoma [8].

Five-year observed survival and median survival time are primarily used to express cancer survival outcomes. Bladder cancer survival studies conducted worldwide produced heterogeneous results. Population-based cancer registries reported five-year observed overall survival between 51% and 59% [9,10]. Meanwhile, cancer registries that exclude non-invasive cases recorded lower survival rates between 33% and 44% [11,12,13]. Within Asian countries, other hospital-based studies found five-year observed survival between 28% and 58% depending on the patients’ criteria [14,15,16]. Median survival time in Scotland and Belgium were 32 and 47 months, respectively [11,12]. When treatments were considered, patients with muscle-invasive cancer receiving radical cystectomy had the best median survival time between 36 and 52 months [17,18,19,20]. Worst median survival times of fewer than four months were found among stage IV patient who did not receive chemotherapy [21,22,23].

Cancer survival is one of the key indicators to represent healthcare performance and monitoring cancer care progress. A hospital-based study in Malaysia mentioned 33 months as the mean survival time among muscle-invasive bladder cancer patients [8]. Since bladder cancer was not included in the Malaysian Study on Cancer Survival (MySCAN) and CONCORD-3 project of Global Surveillance of Trends in Cancer Survival 2000-14 [24,25], our study aimed to describe the characteristics and determine the survival of bladder cancer patient in Malaysia diagnosed between 2007 and 2011 using population-based data.

## 2. Materials and Methods

### 2.1. Study Area

Malaysia is located in Southeast Asia and divided into two main regions; Peninsular Malaysia on mainland Asia and East Malaysia on the northern island of Borneo. Malaysia consisted of 13 states and three federal territories, with over 30 million population [4,25].

### 2.2. Study Design

This is a retrospective study conducted from January to March 2019. Data were obtained from the Malaysian National Cancer Registry, a population-based nationwide coverage cancer registry that received cancer notifications. The registry was established in 2002 and coordinates 15 sub-national cancer registries. The registries received data from multiple sources, including cancer-related pathological, hospital discharge and death records from public and private healthcare facilities to ensure inclusion of all cancer cases [25]. Our study was restricted to Malaysian citizen aged 15-year-old and above with malignant bladder cancer patient (International Classification of Disease for Oncology, 3 edition [ICD-O-3], topographical codes C67) diagnosed between 1 January 2007 and 31 December 2011. In situ and benign tumours were excluded. Vital status was updated until 31 December 2016 with the national death registry based on the unique national identity card number. The registrars extracted demographic (age, sex, and ethnicity) and clinical variables at diagnosis (morphology, stage, surgery, radiotherapy, and chemotherapy status) of bladder cancer records. The data were prepared in a Microsoft Excel file. Duplicate records and cases with an invalid date of diagnosis were excluded. Invalid date of diagnosis refers to incidence date documented after the date of death and incidence date of less than two-week duration from death date for patients recorded to have received radiotherapy and/or chemotherapy with histological confirmation [25].

### 2.3. Statistical Analysis

Data obtained in Microsoft Excel format were imported in SPSS Software version 24 for analysis. Data were explored for missing entry and distribution of numerical data. Five-year observed survival was estimated using the life table method. The five-year observed survival refers to the proportion of alive patients after five years of diagnosis. The Kaplan–Meier survival estimates method was applied to determine median survival time, which is the first observed time when half of the patients died. The outcome of interest in our analysis was time-to-event. The event referred to death regardless of the cause of death. Patients who did not experience the event was considered censored at the end of the study period or the last available follow-up date. Survival time (in months) was calculated from the date of diagnosis to death or censored. Analyses according to the stage were presented as well for a fairer comparison with other studies.

### 2.4. Ethical Approval

The study was conducted according to the principles outlined in the Declaration of Helsinki. Ethical approval was obtained from the Human Research and Ethics Committee, Universiti Sains Malaysia (USM/JEPeM/18100500) and the Medical Review and Ethical Committee, Ministry of Health Malaysia (NMRR-18-2965-44397 (IIR)).

## 3. Results

### 3.1. Characteristics

After excluding duplicate records (*n* = 54) and cases with an invalid date of diagnosis (*n* = 29), we analysed 1,828 bladder cancer patients diagnosed between 2007 and 2011. The mean (SD) age of diagnosis was 64.9 (12.5) years old. The patients were predominantly male (*n* = 1438, 78.7%) and Malay. The most common cancer morphology was transitional cell carcinoma, with 1430 (78.2%) cases. Almost half of the patient received surgical treatment at diagnosis. Meanwhile, more than half of the data regarding the stage at diagnosis, receipt of chemotherapy and radiotherapy at diagnosis were unrecorded. Detailed characteristics can be found in Table 1.

### 3.2. Five-Year Observed Survival

After five years of diagnosis, it is estimated that 36.9% (95% CI: 34.6, 39.1) of bladder cancer patients survived. Table 2 shows the results of five-year observed survival according to the stage. Patients with stage I disease had the highest five-year survival (67.6%, 95% CI: 62.0, 73.3). Survivals markedly deteriorated for stage II (34.3%, 95% CI: 27.9, 40.8), stage III (25.7%, 95% CI: 18.7, 32.6) and stage IV (12.2%, 95% CI: 8.1, 16.3).

### 3.3. Median Survival Time

**Figure 1:**
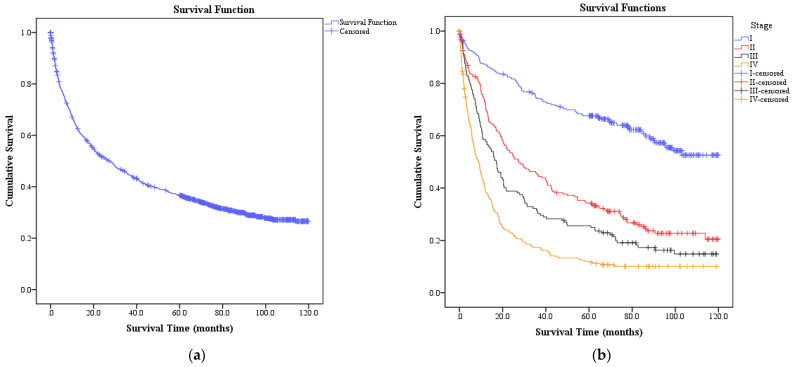
Kaplan–Meier Curve for survival estimate according among bladder cancer patients in Malaysia, i.e., for: (**a**) Overall patients; (**b**) Patients according to the stage at diagnosis.

The median survival times for bladder cancer patients in Malaysia (*n* = 1828) was 27.3 months (95% CI: 23.6, 31.0). Median survival times according to cancer stage are presented in Table 2. No median survival time could be estimated for stage I bladder cancer patients because less than half of the patient died at the end of the study period. The Kaplan–Meier curves for overall and according to the stage at diagnosis are presented in Figure 1. 

## 4. Discussion

In this study, we described the characteristics and estimated the survivals of bladder cancer patients in Malaysia. Our study found that the mean age of diagnosis resembled the previous study in Malaysia [8], other Asian countries [15,16], and European countries [6,26,27]. However, the mean age of diagnosis was younger than documented in the United States, which was more than 70 years old [18,22,23]. Male to female distribution in our study reflected the global distribution and other literature [10,26,28,29]. A disproportionately high prevalence of tobacco smoking and particular carcinogenic occupational exposure among men could explain the striking difference [28,30]. The differences in the biological and hormonal mechanism between sex had also been suggested [31].

The largest ethnic group among Malaysian citizen were Malay, followed by Chinese and Indian [4]. Therefore, our study revealed similar trends. However, a study in Kuala Lumpur, Malaysia, stated that the Chinese make up the largest patient group [8]. The findings might be resulted from limited patients’ enrolment and was conducted in an urban area with a high Chinese population. The percentage of urothelial carcinoma in our study sample was lower than in other studies (range: 86–96%). The result possibly due to a relatively high proportion of nonspecific morphology in our sample could be the urothelial carcinoma histology [6,10,18,32]. 

The proportion of unrecorded bladder cancer stage was higher than the top three cancer in Malaysia. Female breast, colorectal and lung cancer had 32.7%, 42.4% and 41.2% of unrecorded stage, respectively [25]. Stage data were also substantially missing in other studies [16,21]. Population-based cancer registries in Europe faced similar challenges, with only four cancer registries providing a quality cancer stage for further analysis [33]. Among those with a recorded stage at diagnosis, the proportion of stage I was 30.4%, stage II (24.3%), III (17.2%) and IV (28.1%). A similar distribution was found in Indonesia’s national referral centre. In the study, the percentage of stage I, II, III and IV were 27.6%, 24.0%, 19.3% and 19.3%, respectively [15].

In contrast, more than four out of five patients in Norway and the United States presented at the early localised stage [1,2,34]. Stage IV distribution in our sample was two times higher than that of the Netherlands Cancer Registry and Yazd hospitals, Iran [6,16]. Stage IV patients have a grave prognosis, and their higher percentage among study populations decreased overall five-year survival and median survival time.

The overall five-year observed survival for bladder cancer patients in Malaysia was better than a hospital-based study in Jakarta, Indonesia, which reported five-year survival of 27.6% [15]. Yet, in contrast, survival was markedly low compared to the developed countries. The neighbouring country, Singapore, recorded a five-year survival of 58.5% and 53.6% for men and women, respectively [9]. A study in Kanagawa, Japan, among patients diagnosed between 2003 and 2010 found higher five-year survival of 54% and 51% among males and females, respectively [10]. The study includes approximately 10% of urothelial carcinoma in situ, and 95.1% of the patients were transitional cell carcinoma, compared to none and 78.2% in our study. The substantial difference might contribute to lower survival in our study as in situ lesion and urothelial carcinoma associated with better prognosis. Similarly, the five-year survival was 54.5% and 47.8% for male and female bladder cancer patients in New South Wales, Australia [35]. The patient population had a higher proportion of localised disease (49.4%) and transitional cell histology (89.9%). However, our findings were incongruent with other population-based cancer registries which exclude benign lesion in Scotland, Denmark, and Belgium. These registries reported five-year survival between 33.3% and 44.0% [11,12,13].

Regarding the overall median survival time of bladder cancer, our study’s finding was much lower than the findings in Iran of 51 months [16]. However, the study restricted to bladder cancer patients referred to tertiary centres and received radiotherapy and chemotherapy. Patients without treatment at diagnosis fare worse in term of survival and expectedly lowering the overall median survival time [18]. Population-based cancer registries in Scotland (46.8 months) and Belgium (male: 45, female: 32 months) recorded a more favourable median survival time nevertheless [11,12].

Comparing overall five-year survival using population-based cancer registries’ database should be made cautiously, particularly in bladder cancer. Cancer registries in the United States, Europe and the developed countries had included in situ and non-invasive bladder cancer in their database due to the malignant potential and inability to differentiate between in situ and invasive lesion reliably. Registries which omit non-invasive bladder tumour found to have the lowest five-year survival in the European Cancer Registry Based Study on Survival and Care of Cancer Patients, EUROCARE-5 [1,7,36]. Since our bladder cancer patients presented with more adverse disease characteristic such as a higher percentage of advanced stage at diagnosis, exclusion of non-invasive tumour and lower proportion of urothelial carcinoma, it is not surprising that the overall five-year survival was lesser than other studies. Therefore, discussing our survival findings according to the stage at the initial presentation would provide better insight.

For stage I, our five-year survival was better than what was found in Jakarta of 53.8% [15], similar to a study in Yazd, Iran of 66% [16], and lower than 71% recorded in Erlangen, Germany [27]. Median survival time for stage I could not be estimated as more than half of the patient still alive until the closure of the study. Stage II and III in bladder cancer are more or less equivalent to non-metastatic muscle-invasive bladder cancer disease. Several studies, which enrolled these patients who underwent cystectomy surgery, reported overall five-year survival and median survival time of more than 45% and 36 months [14,20,23]. Patients managed by curative intent surgery were known to have an excellent prognosis, thereby explaining higher survival. Our result is comparable to a study in Jakarta [15] and a study that includes all patients regardless of treatment type [19]. Meanwhile, for stage IV, the five-year survival determined by this study was consistent with other studies suggesting inevitable severe prognosis in this subgroup of patients [15,32]. Similarly, our stage IV patient’s median survival time was within the range of the multiple studies conducted worldwide between three and 16 months [21,22,23,26,32].

The limitation of our study primarily resulted from the limitations of using secondary data. A high proportion of unrecorded data in the morphology, stage, surgery, radiotherapy, and chemotherapy variable restricted us to explore the factors contributing to the lower survival result. The strength of our study was using population-based nationwide data. The finding could be generalised to the Malaysian population. Completeness of death status follow-up in our study is another key strength. The registry updated the vital status as provided by the mortality registry using the Malaysian citizens’ identification number.

## 5. Conclusions

Bladder cancer survival among Malaysian was lower with increasing stage of cancer. Among the recorded stages at diagnosis, the overwhelming majority of patients presented at a later stage, which carries a poor survival rate. Data on treatment options were scarce to evaluate current treatment modalities offered. These findings highlight the importance of cancer prevention and early detection. Community and primary care practitioners should be empowered to live healthily and recognise the early signs and symptoms for expedite referral and comprehensive management care. Cancer registration should be strengthened and linked with the healthcare information system for robust cancer care surveillance.

## Figures and Tables

**Table 1:** Bladder cancer patients’ characteristics (*n* = 1828).

Characteristics	*n* (%)
Age (mean, SD)	64.9 (12.5)
Sex	
Male	1438 (78.7)
Female	390 (21.3)
Ethnicity	
Malay	903 (49.4)
Chinese	684 (37.4)
Indian	107 (5.9)
Iban	36 (2.0)
Kadazan	17 (0.9)
Others	81 (4.4)
Morphology	
Urothelial carcinoma	1430 (78.2)
Squamous cell carcinoma	57 (3.1)
Adenocarcinoma	150 (8.2)
Sarcoma	10 (0.6)
Others	7 (0.4)
Nonspecific	174 (9.5)
Stage	
I	270 (14.8)
II	216 (11.8)
III	153 (8.4)
IV	250 (13.7)
Unrecorded	939 (51.4)
Surgery	
Yes	902 (49.3)
No	297 (16.3)
Unrecorded	629 (34.4)
Radiotherapy	
Yes	221 (12.1)
No	573 (31.3)
Unrecorded	1034 (56.6)
Chemotherapy	
Yes	191 (10.5)
No	587 (32.1)
Unrecorded	1050 (57.4)

**Table 2:** Five-year observed survival and median survival time for bladder cancer patients in Malaysia.

Stage	Five-Year Survival % (95% CI)	Median Survival Time Months (95% CI)
I	67.6 (62.0, 73.3)	-
II	34.3 (27.9, 40.8)	27.6 (18.1, 37.0)
III	25.7 (18.7, 32.6)	17.0 (13.3, 20.7)
IV	12.2 (8.1, 16.3)	8.8 (7.0, 10.7)
Unrecorded	36.9 (33.7, 40.1)	29.3 (23.5, 31.0)
All	36.9% (95% CI: 34.6, 39.1)	27.3 (23.6, 31.0)

## Data Availability

The data that support the findings are available from the National Cancer Institute, Ministry of Health Malaysia, but restrictions apply to the availability of these data. These data were used under agreement for the current study and are not publicly available. Data are, however, available from the authors but only with the explicit permission of the Director-General, Ministry of Health Malaysia.
